# Effect of age, sex, and morbidity count on trial attrition: meta-analysis of individual participant level data from phase 3/4 industry funded clinical trials

**DOI:** 10.1136/bmjmed-2022-000217

**Published:** 2022-09-01

**Authors:** Jennifer S Lees, Peter Hanlon, Elaine W Butterly, Sarah H Wild, Frances S Mair, Rod S Taylor, Bruce Guthrie, Katie Gillies, Sofia Dias, Nicky J Welton, David A McAllister

**Affiliations:** 1 University of Glasgow, Glasgow, UK; 2 Institute of Health and Wellbeing, University of Glasgow, Glasgow, UK; 3 University of Edinburgh, Edinburgh, UK; 4 University of Aberdeen, Aberdeen, UK; 5 University of York, York, UK; 6 Population Health Sciences, University of Bristol, Bristol, UK

**Keywords:** clinical trial, internal medicine, medicine, research design

## Abstract

**Objectives:**

To estimate the association between individual participant characteristics and attrition from randomised controlled trials.

**Design:**

Meta-analysis of individual participant level data (IPD).

**Data sources:**

Clinical trial repositories (Clinical Study Data Request and Yale University Open Data Access).

**Eligibility criteria for selecting studies:**

Eligible phase 3 or 4 trials identified according to prespecified criteria (PROSPERO CRD42018048202).

**Main outcome measures:**

Association between comorbidity count (identified using medical history or concomitant drug treatment data) and trial attrition (failure for any reason to complete the final trial visit), estimated in logistic regression models and adjusted for age and sex. Estimates were meta-analysed in bayesian linear models, with partial pooling across index conditions and drug classes.

**Results:**

In 92 trials across 20 index conditions and 17 drug classes, the mean comorbidity count ranged from 0.3 to 2.7. Neither age nor sex was clearly associated with attrition (odds ratio 1.04, 95% credible interval 0.98 to 1.11; and 0.99, 0.93 to 1.05, respectively). However, comorbidity count was associated with trial attrition (odds ratio per additional comorbidity 1.11, 95% credible interval 1.07 to 1.14). No evidence of non-linearity (assessed via a second order polynomial) was seen in the association between comorbidity count and trial attrition, with minimal variation across drug classes and index conditions. At a trial level, an increase in participant comorbidity count has a minor impact on attrition: for a notional trial with high level of attrition in individuals without comorbidity, doubling the mean comorbidity count from 1 to 2 translates to an increase in trial attrition from 29% to 31%.

**Conclusions:**

Increased comorbidity count, irrespective of age and sex, is associated with a modest increased odds of participant attrition. The benefit of increased generalisability of including participants with multimorbidity seems likely to outweigh the disadvantages of increased attrition.

**WHAT IS ALREADY KNOWN ON THIS TOPIC**

- In the general population, many patients have multimorbidity
- In clinical trials, participants with multimorbidity are under-represented
- This under-representation might partly be driven by concerns that participants with multimorbidity will find participation challenging, leading to high levels of attrition, imposing additional costs and burdens on trial conduct, and potentially compromising validity without increasing representativeness

**WHAT THIS STUDY ADDS**

- Individuals with multimorbidity are less likely to complete participation in clinical trials, but the effect is modest
- Men were not evidently less likely than women to complete participation in clinical trials, nor were older people than younger people, but data suggested an association between increasing age and attrition

**HOW THIS STUDY MIGHT AFFECT RESEARCH, PRACTICE, OR POLICY**

- Improving representation of patients with multimorbidity in trials and improving trial validity is feasible, without prohibitive increases in attrition

## Introduction

Comorbidity occurs in around half of people with any long term condition; it is increasing in prevalence but is substantially less common in participants included in randomised controlled trials.^1 2^ Where participants with comorbidity are under-represented within trials, the applicability of trials treatment effect estimates are uncertain, particularly for absolute treatment effects.^3^ Consequently, increased recruitment of participants with comorbidity might be desirable. However, one potential disincentive to enrolling more people with comorbidities is the concern that they might be less able to complete the trial,^4 5^ leading to increased participant attrition.

Even when similar across treatment arms, high levels of attrition lead to a reduction of the precision with which treatment effects can be estimated for a given sample size,^6^ raise concerns about the interpretation of intention to treat estimates^7^ (as participants who no longer participate are unlikely to follow trial treatment protocols), can make trials less representative (as participants who leave the study could be different from those who complete the study),^8^ and might cause a loss of confidence among researchers reviewing studies since high attrition could be perceived as a marker of suboptimal trial conduct.

Although methods to improve the retention of participants have been extensively studied,^9–12^ we are not aware of any study that has quantified the association between comorbidity and trial attrition. Such estimates would be valuable for informing trial design (by informing sample size calculations) and conduct (by identifying those individuals most likely to withdraw or be lost to follow-up, and devising and testing strategies to improve their retention). In a previously published meta-analysis of individual participant level data (IPD) of 92 standard phase 3 or 4 industry funded trials, we reported that multimorbidity (and hence comorbidity) was present in trials, although at a lower prevalence than found in the community.^13^ In the present IPD meta-analysis of this same set of trials, we aimed to determine whether comorbidity, age, and sex are associated with attrition among trial participants.

## Methods

### Study design

We performed a meta-analysis of trial IPD to determine the association between comorbidity, age, and sex on attrition, in two stages. Firstly, for each trial, the association between comorbidity count (the number of other conditions in addition to the index condition defining the trial population) and attrition (defined as failure for any reason to complete final trial visit) was estimated in logistic regression models, adjusting for age and sex. In similar models, we estimated the associations between age and sex and trial attrition. Secondly, the resulting effect estimates were meta-analysed in bayesian linear models. We allowed partial pooling across index conditions and drug classes in order to obtain overall, drug class specific and index condition specific estimates of these associations.

### Data sources and participants

Available IPD were obtained from phase 3 or 4 trials contained within two trial repositories: the multi-sponsored Clinical Study Data Request repository and the Yale University Open Data Access project. Appropriate trials for inclusion were identified according to prespecified criteria (PROSPERO CRD42018048202).^13^ Specifically, we included trials for medical conditions that are predominantly managed by drug treatments (frequently over a sustained period).^13^

We classified each trial in terms of the index condition based on the stated trial indication as described previously.^13^ Each trial was also classified in terms of the intervention drug, using the five character WHO Anatomic Therapeutic Chemical (ATC) class.^14^ For example, the A10BJ (glucagon-like peptide 1 analogues) class includes the drugs A10BJ01 (exenatide) and A10BJ02 (liraglutide).

In a previous publication,^13^ we defined comorbidities solely using concomitant drug treatments in order to enable comparison across trial and community settings. The comorbid conditions included cardiovascular disease, chronic pain, arthritis, affective disorders, acid related disorders, asthma or chronic obstructive pulmonary disease, diabetes mellitus, osteoporosis, thyroid disease, thromboembolic disease, inflammatory conditions, benign prostatic hyperplasia, gout, glaucoma, urinary incontinence, erectile dysfunction, psychotic disorders, epilepsy, migraine, parkinsonism, and dementia. For the current analysis, for the 80 trials that did not redact medical history data, we additionally defined the same comorbidities using prespecified codes from the Medical Dictionary for Regulatory Activities. Individuals were defined as having a comorbidity if they met required definitions based on either concomitant drug treatment or medical history. Definitions and code lists are available at the project repository^15^ (https://github.com/ChronicDiseaseEpi/como_complete_public). To produce a comorbidity count for each trial participant, the number of comorbidities at baseline were summed, excluding the index condition of the respective trials.

### Outcomes

The outcome of interest was attrition, defined as failure for any reason to complete the final trial visit, including intentional and non-intentional withdrawals. The proportion of attritions was estimated as the number of participants who did not complete as a proportion of all those randomised.

### Representativeness

Not all sponsors share trial IPD and not all trials are made available to third party researchers. Consequently, to contextualise the IPD trials included in this analysis, we also examined attrition in a wider set of trials registered on the US clinical trials registry (ClinicalTrials.gov) of which the IPD trials are a subset (PROSPERO registration number CRD42018048202).^13^ We restricted the 2235 trials registered on ClinicalTrials.gov to the 777 registered on or after 2010 since we saw that trials registered before this period (consistent with changes in US Food and Drug Administration requirements for trials registered on or after 2007)^16^ were less likely to post completion data. Of these, 593 (76.3%) trials had posted data to ClinicalTrials.gov on enrolment, randomisation, and completion, for which we produced summaries of the proportion of participants completing each trial overall and by index condition.

### Statistical analysis

Summary statistics were calculated for each index condition for the available IPD trials including age (mean and standard deviation), sex (number and %), comorbidity count (mean and standard deviation), and proportion with two or more comorbidities. A violin plot was constructed to illustrate the proportion of attritions in IPD and ClinicalTrials.gov trials.

Full descriptions of the modelling are provided in the online supplemental appendix and are described briefly below. In logistic regression models, for each trial, attrition was regressed on age (per 15 year increment, which was close to the standard deviation for most trials), sex (male *v* female (reference)), and comorbidity count (per additional comorbidity). We fitted a range of models with and without terms for comorbidity count, comorbidity count squared, age, sex, treatment arm, and a comorbidity-treatment arm interaction. The effect measure estimates (log-odds ratios) and associated standard errors for each model were then exported from the Yale University Open Data Access and Clinical Study Data Request repository safe havens. Proportions of missing baseline data within trials were very small. Logistic regression models within trial repositories were conducted on complete cases.

10.1136/bmjmed-2022-000217.supp1 Supplementary data

The effect measure estimates for the age (adjusted for sex), sex (adjusted for age), and comorbidity (adjusted for age and sex) terms were subsequently meta-analysed separately in bayesian linear regression models. We used bayesian models because these allowed partial pooling across index conditions and drug classes and because they allowed us to obtain credible intervals for estimates at the level of index conditions and drug classes directly from the posterior without a need for post hoc calculations. We performed a range of meta-analyses for each regression coefficient. These meta-analyses were done within a bayesian framework, where the final meta-analysed estimate was a summary of the trial level estimates. This summary is a product of the precision with which the association is estimated for each trial (ie, the inverse of the squared standard error for the relevant coefficient), the variation between trials, the variation between other groups (eg, drug class or condition), and the prior distributions (a vague prior for the overall effect, and weakly informative priors for the variation parameters). Details of the selected priors are available in the online supplemental data file. For the simplest model, only variation between trials was explicitly modelled. For the (progressively) more complex models, the variation between other groups was also modelled: drug class, condition, and both drug class and condition. This modelling allowed estimates to differ for each group, while also allowing sharing of information between the groups (known as partial pooling), which has the effect of improving precision as well as shifting extreme effect estimates towards the overall mean. The variation within groups for trials, conditions, and drug classes was reported as the respective standard deviation.

**Figure 1:**
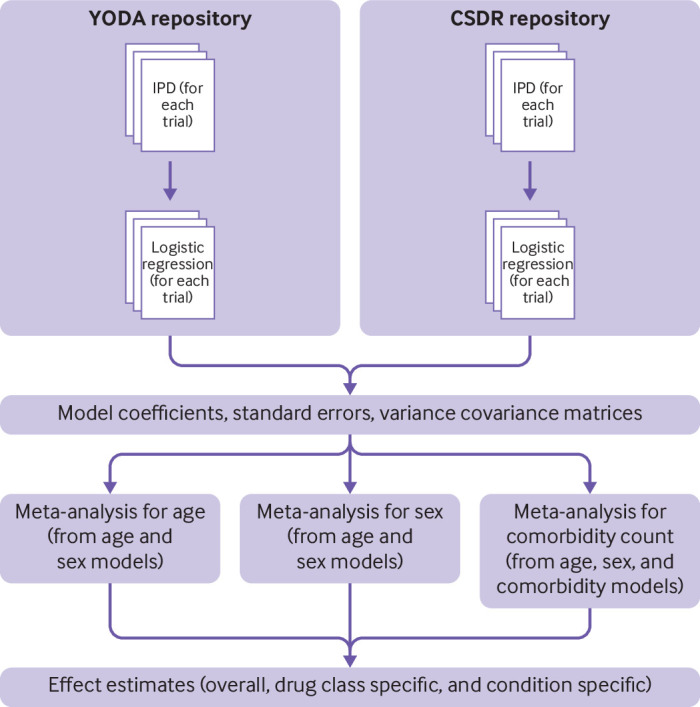
Overview of use of models for meta-analysis output. Shaded areas=analyses conducted within Clinical Study Data Request (CSDR) and Yale University Open Data Access (YODA) repository safe havens. Variance matrices of the effect estimates were exported to allow maximum flexibility in subsequent meta-analyses, if required. IPD=individual participant level data

Models were fit using the brms package.^17^ For each model, 4000 samples from the posterior were obtained and summarised as 50%, 80%, and 95% credible intervals. The probability (bayesian P value) that comorbidity count was positively associated with attrition was estimated as the proportion of the posterior distribution of the log-odds ratio, which was above 0. An illustration of models used to assess the association between comorbidity count and attrition is displayed in figure 1.

Using the effect estimates obtained from this meta-analysis for the association between comorbidity count and attrition among participants, we then explored the potential impact of comorbidity count at the trial level. Firstly, we constructed a set of notional trials with different plausible mean comorbidity counts (and therefore with different proportions of participants with each comorbidity count) and different risks of attrition among participants with zero comorbidities (which could differ because of trial level factors such as difference in follow-up methods or settings). Next, we applied the effect estimates to participants from these notional trials to estimate the overall percentage of participants who would be expected not to complete the trial visits. This analysis is described in detail in the online supplemental appendix.

We conducted a sensitivity analysis using wider priors for the variances between trials, between conditions, and between drug classes (details in online supplemental data file). We also conducted a sensitivity analysis within each of the trial repositories where we reanalysed the trials having excluded any participant who had an adverse event of any kind. The model outputs for these trials were not exported but were meta-analysed within the repositories, pooling results across all trials. We fit a frequentist random effects model (which assumes effect estimates for each trial come from a normal distribution), using a restricted maximum likelihood estimator within the metafor package. This model was fit using frequentist software rather than the bayesian software used for the main analysis, because bayesian software was not available within the trial repository. The trial level results, model outputs, and analysis code are provided on the project GitHub repository (https://github.com/ChronicDiseaseEpi/como_complete_public).

### Patient and public involvement

Patients or the public were not involved in the design, or conduct, or reporting of this study, but will be involved in dissemination plans of this research. Refer to the methods section for further details.

## Results

### Included trials

A total of 92 IPD trials featured 20 index conditions trialling drugs from 17 ATC drug classes. The index conditions with the most trials were type 2 diabetes mellitus (18 trials), inflammatory bowel disease (10 trials), rheumatoid arthritis (10 trials), and hypertension (nine trials).

### Baseline characteristics

The age and sex distribution for IPD trials differed by condition (table 1). In trials for atrial fibrillation and dementia, the mean age was over 70 years; in trials for migraine and inflammatory bowel disease, the mean age was under 40 years. Trials ranged from those conducted solely in men (prostate disease, 100% men) to those predominantly conducted among women (migraine, 85.4% women).

**Table 1:** Baseline characteristics of study trials and participants

Index condition	No (%) of trials	Mean (SD) age (years)	Sex (No (%))	Trial attrition (proportion)	Mean (SD) comorbidity count	>2 comorbidities (proportion)
Atrial fibrillation	1	71.7 (8.4)	Female (6554 (36.3))	0.11	1.1 (0.0)	0.29
			Male (11 479 (63.7))	0.11		
Axial spondyloarthritis	2	41.4 (11.7)	Female (91 (28.4))	0.05	0.9 (0.1)	0.22
			Male (229 (71.6))	0.02		
Benign prostatic hyperplasia	6	63.4 (8.5)	Female	—	1.2 (0.7)	0.37
			Male (2816 (100.0))	0.06		
Chronic idiopathic urticaria	3	42.6 (14.1)	Female (719 (73.5))	0.14	1.6 (0.6)	0.48
			Male (259 (26.5))	0.14		
Dementia (any)	3	74.2 (7.8)	Female (1473 (59.6))	0.30	1.8 (0.4)	0.56
			Male (999 (40.4))	0.26		
Type 2 diabetes mellitus	18	57.3 (9.3)	Female (8091 (43.4))	0.24	1.1 (0.5)	0.29
			Male (10 559 (56.6))	0.23		
Hypertension	9	54.5 (15.6)	Female (2202 (42.7))	0.12	0.6 (0.3)	0.12
			Male (2949 (57.3))	0.12		
Pulmonary hypertension	1	54.5 (15.6)	Female (318 (78.3))	0.13	2.1 (0)	0.58
			Male (88 (21.7))	0.13		
Inflammatory bowel disease	10	38.5 (12.6)	Female (2272 (49.3))	0.19	0.8 (0.2)	0.20
			Male (2336 (50.7))	0.20		
Knee arthroplasty	1	66.1 (9.5)	Female (1494 (57.6))	0.07	2.5 (0)	0.65
			Male (1099 (42.4))	0.07		
Migraine	5	39.1 (12.3)	Female (1250 (85.4))	0.25	1 (0.6)	0.21
			Male (214 (14.6))	0.27		
Osteoarthritis	1	63.7 (11.7)	Female (888 (67.3))	0.11	1.8 (0)	0.54
			Male (432 (32.7))	0.11		
Osteoporosis	1	56.5 (13.9)	Female (345 (80.4))	0.44	2.7 (0)	0.84
			Male (84 (19.6))	0.44		
Parkinson’s disease (all)	3	61.7 (10)	Female 577 (42.2)	0.12	1.4 (0.6)	0.41
			Male (791 (57.8))	0.12		
Psoriasis	4	45 (12.8)	Female (836 (30.8))	0.01	0.3 (0.2)	0.70
			Male (1878 (39.2))	0.01		
Psoriatic arthropathy	3	47.5 (11.5)	Female (266 (44.4))	0.14	0.5 (0.4)	0.14
			Male (333 (55.6))	0.10		
Pulmonary fibrosis	2	66.9 (8.1)	Female (220 (20.7))	0.17	0.4 (0.1)	0.09
			Male (843 (79.3))	0.17		
Restless legs syndrome	2	53.9 (12.7)	Female (414 (61.2))	0.24	1.6 (0.2)	0.50
			Male (262 (38.8))	0.24		
Rheumatoid arthritis	10	52.3 (12)	Female (4507 (80.2))	0.21	1.3 (0.4)	0.34
			Male (1111 (19.8))	0.21		
Thromboembolism	7	57.8 (13.7)	Female (8106 (44.8))	0.13	1.1 (0.5)	0.32
			Male (9985 (55.2))	0.13		

SD=standard deviation.

For each trial, the comorbidity count was summarised well with a Poisson distribution (figure S1). The mean comorbidity count across index conditions ranged from 0.3 for psoriasis to 2.7 for osteoporosis (table 1). For many trials, a substantial proportion of participants had two or more comorbidities in addition to the index disease, for example, in trials of dementia (56%; n=3 trials), osteoarthritis (54%; n=1 trial), Parkinson’s disease (41%; n=3 trials), and type 2 diabetes (29%; n=20 trials); but few participants had two or more comorbidities in hypertension trials (12%; n=9 trials) or pulmonary fibrosis trials (9%; n=2 trials).

### Trial attrition and age, sex, and comorbidity count

**Figure 2:**
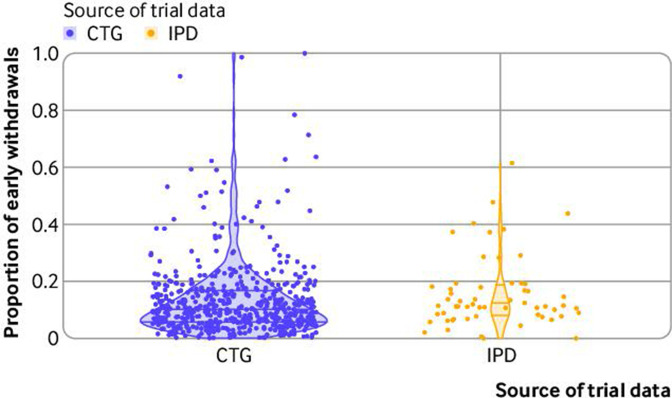
Plots of the proportion of attritions for 593 clinical trials registered on ClinicalTrials.gov (CTG) and 92 trials with individual participant level data (IPD) available. Violin plots represent the summary estimates for CTG and IPD trials. Horizontal bars=median and interquartile ranges; dots=proportion of attrition for individual trials

We saw substantial variation in attrition in the IPD trials both within and between index conditions (table 1 and figure 2).

No clear association was seen between increasing age and attrition (table 2); the odds ratio for attrition per 15 year increment in age was 1.04 (95% credible interval 0.98 to 1.11), although the probability of a positive association (bayesian P value) was 89% (table 2). We saw no clear association between sex and attrition (odds ratio 0.99, 95% credible interval 0.93 to 1.05; table 2).

**Table 2:** Odds ratios for association between trial attrition and age, sex, and comorbidity count for different models

Model‡	Comorbidity count*			Age†			Sex†		
	No	Odds ratio (95% CrI)	Bayesian P value	No	Odds ratio (95% CrI)	Bayesian P value	No	Odds ratio (95% CrI)	Bayesian P value
Pooled (ignoring drug class and index condition)	n=89 trials	1.11 (1.07 to 1.14)	1.00	n=90 trials	1.04 (0.98 to 1.11)	0.89	n=83 trials	0.99 (0.93 to 1.05)	0.32
SD trial	0.10	—	—	0.22	—	—	0.13	—	—
Partial pooling across index conditions	n=20 index conditions	1.11 (1.06 to 1.15)	1.00	n=20 index conditions	1.06 (0.97 to 1.20)	0.89	n=18 index conditions	0.99 (0.90 to 1.11)	0.41
SD trial	0.09	—	—	0.21	—	—	0.11	—	—
SD index condition	0.05	—	—	0.09	—	—	0.12	—	—
Partial pooling across drug classes	n=17 drug classes	1.09 (1.05 to 1.15)	1.00	n=17 drug classes	1.04 (0.97 to 1.13)	0.86	n=17 drug classes	1.00 (0.92 to 1.11)	0.50
SD trial	0.10	—	—	0.22	—	—	0.09	—	—
SD drug class	0.04	—	—	0.07	—	—	0.11	—	—
Partial pooling across index conditions and drug class	n=20 index conditions; n=17 drug classes	1.11 (1.05 to 1.15)	1.00	n=20 index conditions; n=17 drug classes	1.05 (0.96 to 1.16)	0.89	n=18 index conditions; n=17 drug classes	1.00 (0.90 to 1.12)	0.49
SD trial	0.09	—	—	0.21	—	—	0.09	—	—
SD index condition	0.04	—	—	0.09	—	—	0.09	—	—
SD drug class	0.04	—	—	0.07	—	—	0.09	—	—

CrI=credible interval; SD=standard deviation.

*Trial level models adjusted for age, sex, and comorbidity count.

†Trial level models adjusted for age and sex. Bayesian P value to describe proportion of distribution above odds ratio of 1. See online supplemental appendix for full description of models.

‡Standard deviation for variation within groups for trials, index conditions, and drug classes on log-odds scale.

By contrast, comorbidity count was associated with attrition independently of age and sex (odds ratio per additional comorbidity 1.11, 95% credible interval 1.07 to 1.14). We saw no evidence of departure from linearity (estimated via the addition of a squared term to the model) for this association (1.01-fold difference, 95% credible interval 1.00 to 1.03, in odds ratio for increment in comorbidity count of 0 to 1 *v* odds ratio for increment in comorbidity count from 1 to 2). Compared with individuals with zero comorbidities, the odds ratios are 1.11 (95% credible interval 1.07 to 1.14), 1.23 (1.14 to 1.30), and 1.37 (1.23 to 1.48) in those individuals with one, two, and three comorbidities respectively. Assuming a risk of attrition in individuals with no comorbidities (referred to as underlying risk) of 10%, these odds ratios translate to risks of trial attrition of 11.0% (95% credible interval 10.6 to 11.2%), 12.0% (11.3 to 12.6%) and 13.2% (12.0 to 14.1%) in those individuals with one, two, and three comorbidities, respectively. The results were similar in the simplest models, where all trial results were pooled, as well as in more complex models where trial results were partially pooled within drug classes and index conditions (table 2).

For the association between comorbidity count and attrition, we observed roughly twice as much variation between trials as between index conditions and between drug classes (standard deviation (on log-odds scale) 0.10, 0.05, and 0.04 respectively; table 2).

Table 3 illustrates the potential impact of these findings for the association between comorbidity count and attrition using a notional set of trials, under different assumptions about the trial mean comorbidity count and underlying risks of attrition. Except where the underlying risk was very high, the difference across trials with different mean comorbidity counts was modest. For trials with an underlying risk of 49%, the proportion of participants expected not to complete was 50% for a trial with a mean comorbidity count of 0.5, and 53% for a trial with a mean comorbidity count of 2.

**Table 3:** Risk of attrition for trial by mean trial comorbidity count, according to risk of attrition in those individuals with no comorbidities

Baseline risk (%) of trial attrition (ie, assumed risk in those individuals with no comorbidities)	Mean comorbidity count in trial (% with two or more comorbidities)				
	0.5 (1%)	0.75	1 (8%)	1.25	2 (32%)
5	6	6	6	6	7
15	17	18	18	18	20
25	28	29	29	30	31
35	39	39	40	41	42
45	49	50	50	51	53

Estimated proportion of trial participants likely to withdraw early based on the probability of termination among individuals with no comorbidities and the mean comorbidity count. Assumes odds ratio of 1.1 for attrition per one unit increment in comorbidity count (see results) and that the comorbidity count is Poisson distributed. See online supplemental appendix for detailed steps.

### Variation in associations between trial attrition across index conditions

**Figure 3:**
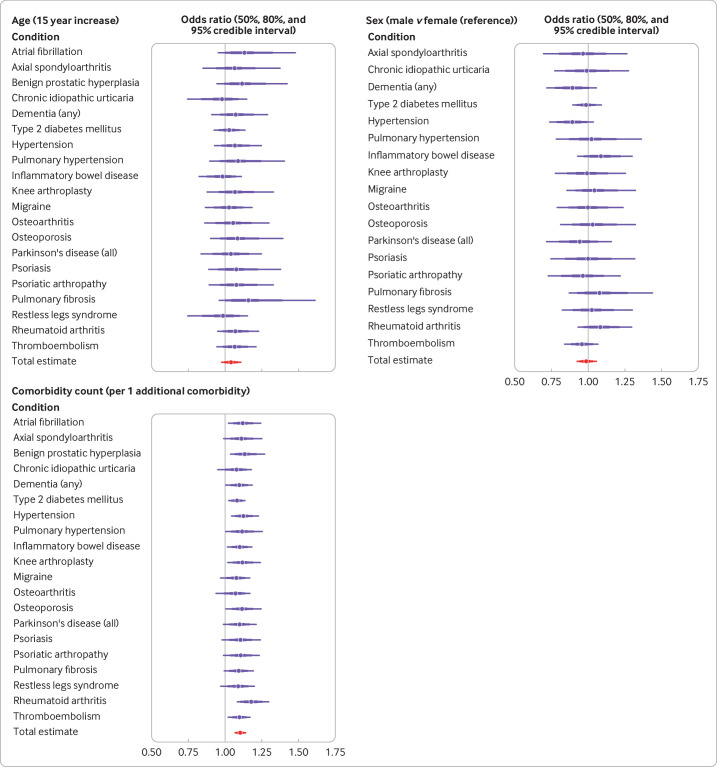
Forest plots showing mean odds ratio of atrial attrition (with 50%, 80%, and 95% credible intervals) by index condition and overall result from pooled model, according to age, sex, and comorbidity count at baseline assessment. Vertical line=reference line (ie, no effect, odds ratio of 1); 50% and 80% credible intervals are shown to indicate that the probability of a given estimate is not uniform across the 95% interval

For the associations between attrition and age, sex, and comorbidity count, we saw little variation across index conditions (figure 3, online supplemental table S1). For age, the strongest association was for pulmonary fibrosis (odds ratio 1.15, 95% credible interval 0.96 to 1.54) and the weakest association was for chronic idiopathic urticaria (0.98, 0.74 to 1.14). For sex, the strongest association was for inflammatory bowel disease (1.08, 0.93 to 1.30) and the weakest association was for psoriasis (1.00, 0.76 to 1.30); for comorbidity count, the strongest association was for rheumatoid arthritis (1.17, 1.08 to 1.29) and the weakest association was for osteoarthritis (1.07, 0.95 to 1.17).

### Variation in associations between trial attrition across drug classes

**Figure 4:**
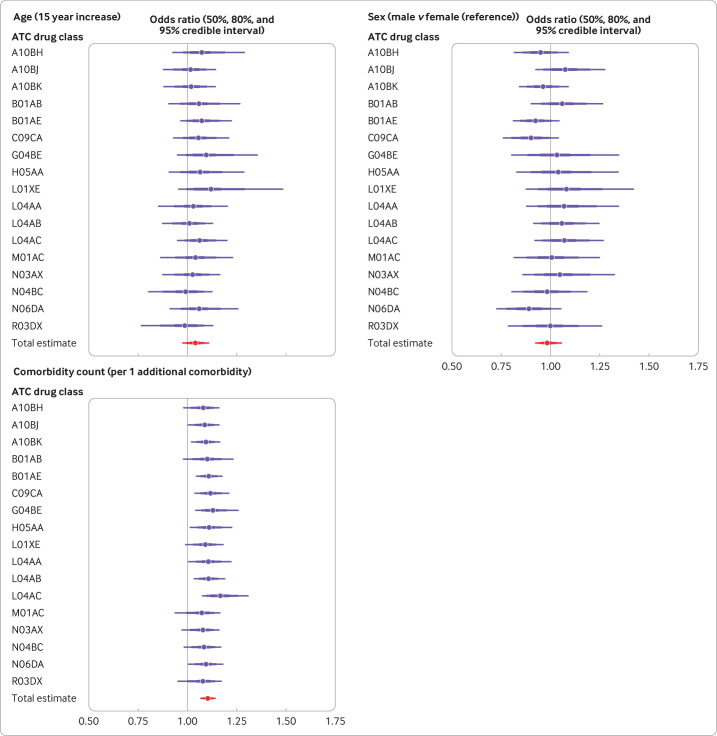
Forest plots showing mean odds ratio of atrial attrition (with 50%, 80%, and 95% credible intervals) by Anatomical Therapeutic Chemical (ATC) drug class and overall result from pooled model, according to age, sex, and comorbidity count at baseline assessment. Vertical line=reference line (ie, no effect, odds ratio of 1); 50% and 80% credible intervals are shown to indicate that the probability of a given estimate is not uniform across the 95% interval

The findings for variations in the associations between trial attrition and age, sex, and comorbidity count by drug classes were similar to those for index conditions (figure 4, online supplemental table S2). For age, the strongest association was for protein kinase inhibitors (odds ratio 1.11, 95% credible interval 0.96 to 1.41) and the weakest association was for dopamine agonists (1.00, 0.82 to 1.13). For sex, the strongest association was for glucagon-like peptide 1 analogues (1.08, 0.93 to 1.28) and the weakest association was for anticholinesterases (0.89, 0.73 to 1.05). For comorbidity count, the strongest association was for interleukin inhibitors (1.17, 1.08 to 1.30) and the weakest association was for oxicams (1.07, 0.93 to 1.17).

### Sensitivity analyses

Sensitivity analyses using wider priors for the variance parameters gave the same results to two decimal places for the variances between trials, between index conditions, and between drug class (online supplemental table S3).

In the frequentist meta-analysis conducted within the Yale University Open Data Access and Clinical Study Data Request repositories where participants with any adverse event were excluded, the associations between comorbidity count and trial attrition were similar to the results from the main analysis (odds ratios 1.08 (95% credible interval 1.03 to 1.13) and 1.13 (1.05 to 1.21) for Yale University Open Data Access and Clinical Study Data Request, respectively).

## Discussion

### Principal findings

In more than 90 trials including more than 90 000 participants across 20 index conditions and 17 drug classes, we found that comorbidity was associated with trial attrition. Neither age nor sex was associated with attrition. The association for comorbidity was moderate, with a 1.1-fold increase in risk per each additional comorbidity after adjusting for age and sex. These findings were consistent across a wide range of index conditions and drug classes.

### Strengths and weaknesses of the study

The strengths of this study include the fact that we analysed IPD for a large number of trials across a range of index conditions and drug classes. However, the study had several limitations. Firstly, the trials were not designed to study comorbidity, and as such our definitions were based on data collected for other reasons (and not redacted for privacy reasons when the IPD was shared): the medical and concomitant drug treatment history collected at baseline. For this reason, we defined comorbidities broadly (eg, asthma and chronic obstructive pulmonary disease were combined into one category) and are likely to have missed some diagnoses. Similarly, patients with comorbidities are likely to take many drug treatments (ie, polypharmacy) across multiple drug classes, or indeed be prescribed one drug for multiple indications. Defining comorbidity counts from drug treatments might have biased associations between comorbidity count and attrition, most likely towards the null because of non-differential misclassification. Although we did not find any evidence for non-linearity in the association between comorbidity count and attrition, we would also caution against extrapolating the findings to comorbidity counts above 3, owing to the very few individuals in the trial with comorbidity counts at this level.

Secondly, although we have showed a clear association between increasing comorbidity count and trial attrition, we cannot be certain that the increased burden of comorbidity was the cause of attrition. An alternative explanation could be unmeasured confounding by other factors that might affect likelihood of trial completion (eg, education, ethnic origin, and socioeconomic status), and we cannot comment on potential mediators of the observed association. Thirdly, we chose to explore non-linearity using a second order polynomial (ie, linear and squared terms for comorbidity count) because this was simpler to implement than other approaches such as splines or fractional polynomials, since we were analysing data across multiple repositories rather than on a single platform. Had we been able to use these more flexible approaches, we might have detected non-linearity not apparent with the current method.

Fourthly, this set of trials was not representative. Trials investigating cancer, infections, psychiatric disorders, and developmental disorders were excluded from the initial search for IPD trials, and among included conditions, we only obtained IPD if the sponsors participated in the data sharing repositories of Clinical Study Data Request or Yale University Open Data Access trials. Moreover, not all trials for sponsors that make data available in this platform were available at the time of our analysis, which meant that the included trials were fairly typical industry funded trials of novel drugs. Nonetheless, we did find that attrition was similarly distributed in IPD trials compared with a wider set of trials included in the US trials register (clinicaltrials.gov). Moreover, the associations within the IPD trials were very consistent across index conditions and ATC drug classes.

### Strengths and weaknesses in relation to other studies

Identifying strategies to improve trial retention is an important research priority,^18^ and several randomised clinical trials have investigated different mechanisms to improve retention.^12^ Participant level factors have been found to be associated with attrition in isolated studies, and include patient preference,^19^ educational status,^20^ poor physical health,^21^ male sex,^22^ and older age.^23^ However, we are not aware of any study that has estimated the strength of the association between individual participant characteristics and attrition across a range of trials. Our study therefore adds to the literature by showing that comorbidity count is associated with attrition, but that (chronological) age and sex do not.

### Meaning of the study

We have previously shown that community populations have about double the comorbidity counts of participants in clinical trials with the same index condition.^13^ In this study, we demonstrated that doubling the comorbidity count in the trial populations was generally associated with an expected absolute increase in attrition of less than 5%, assuming plausible levels of mean trial comorbidity counts and plausible levels of attrition for individuals without comorbidity. If people with a given index condition and comorbidity count in clinical trials and routine clinical practice are similar (an assumption which requires further study), more participants could be included in trials with comorbidity without substantially increasing costs, or compromising the actual or perceived validity of the trial treatment effect estimates.

### Unanswered questions and future research

Improving the representativeness of trial populations first requires a more granular understanding of the factors affecting the retention of participants with higher levels of comorbidity. Among trial participants who did not experience an adverse event, we demonstrated a similar association between comorbidity count and risk of trial attrition as observed in the main analysis, suggesting that attrition in participants with comorbidity is not solely due to an increased risk of adverse events. The mechanisms might alternatively include increased physiological, psychological, or social difficulties in managing the burden of trial participation^19 24^; however, whether attrition is related to intentional (an active decision to withdraw or deliberate non-attendance) or non-intentional withdrawal (where declines in physical or cognitive health preclude further participation) is not clear.

Two specific, potentially measurable indices of physiological wellbeing might be associated with attrition and warrant further exploration. Increasing biological age (eg, measured by DNA methylation)^25^ is associated with the accrual of comorbidity and predicts a variety of morbidity and mortality outcomes.^26^ Accounting for biological (rather than chronological) age could attenuate the association observed between comorbidity count and attrition; however, DNA methylation is not currently routinely measured or reported. Frailty is a marker of functional status that positively correlated with increasing (chronological and biological) age and comorbidity count, but is strongly and independently associated with mortality.^27^ Availability of validated tools to assess frailty (such as Fried^28^ or Rockwood^29^ indices) allow simple recording of these data. Although some trials (especially in disease processes common in older patients, such as dementia) do record participant frailty, the lack of uniform availability of these data from existing trial IPD prevents detailed assessment of the association between frailty and trial attrition. Assessing the impact of frailty on attrition is an important avenue for further study, and particularly whether trial design could be adapted to improve inclusiveness of frailer participants. Specifically, it would be useful to examine whether associations differ according to trial characteristics that might improve completeness of follow-up, such as the use of wearables^30^ or collection of routine data^31 32^ to measure trial endpoints.

Involvement of representative patient and public involvement and engagement (PPIE) groups can help trialists gain a much richer appreciation of the factors driving attrition in trials. With detailed PPIE support at the point of inception, trialists might be better able to design and conduct trials that are likely to be realistic and acceptable to participants with comorbidities. Ultimately, this input could improve recruitment and retention, and drive improvement in outcomes.^33^ Our findings suggest that including people with multimorbidity in such groups is important.

Wider discussions about improving access to clinical trials for under-represented groups (eg, relating to ethnic origin^34 35^ and socioeconomic status) have taken place.^36^ Clinicians might be less likely to approach patients with comorbidities for inclusion owing to concerns that these individuals might be unable to complete the trial.^37^ Importantly, people from more socioeconomically disadvantaged backgrounds could be at higher risk of comorbidity,^38 39^ and particular comorbidities might be more prevalent among people from different ancestries.^39 40^ Consideration should be taken on optimising the inclusion of participants with comorbidities in trials alongside wider considerations of improving access for under-represented groups.

In conclusion, comorbidity count, but not age and sex, is associated with an increased odds of attrition from trials. Although this effect seems modest, it will still involve added costs and time to clinical trials that researchers and funders need to consider. For levels of multimorbidity and attrition typically seen in standard industry funded trials, increasing levels of multimorbidity might be feasible without causing large falls in participant completion.

## Data Availability

Data are available in a public, open access repository. Data may be obtained from a third party and are not publicly available. Individual patient level data are available from Clinical Study Data Request and Yale University Open Data Access platforms. Trial level results, model outputs, and analysis code are provided on the project GitHub repository (https://github.com/ChronicDiseaseEpi/como_complete_public).
