# Supplementary material for: Effect of age, sex, and morbidity count on trial attrition: meta-analysis of individual participant level data from phase 3/4 industry funded clinical trials
Source: BMJ Med. 2022 Sep 1;1(1):e000217. doi: 10.1136/bmjmed-2022-000217 (PMC9978693; doi:10.1136/bmjmed-2022-000217)
Supplement: Supplementary data [file bmjmed-2022-000217supp001.pdf]

Supplementary data

Table S1 Odds ratio for association between attrition and age, sex and comorbidity count by index condition

Index condition	Comorbidity count[1]	Age[2]	Sex[2]
Atrial Fibrillation	1.12 (1.02-1.24)	1.14 (0.95-1.51)	No sex model
Axial Spondyloarthritis	1.11 (0.99-1.27)	1.06 (0.83-1.40)	0.97 (0.70-1.26)
Benign Prostatic Hyperplasia	1.13 (1.04-1.27)	1.13 (0.95-1.48)	No sex model
Chronic idiopathic urticaria	1.08 (0.95-1.18)	0.97 (0.73-1.15)	0.99 (0.77-1.25)
Dementia (any)	1.10 (1.01-1.19)	1.08 (0.90-1.32)	0.89 (0.71-1.06)
Diabetes Mellitus, Type 2	1.08 (1.02-1.14)	1.02 (0.90-1.13)	0.99 (0.90-1.09)
Hypertension	1.12 (1.05-1.22)	1.07 (0.93-1.25)	0.89 (0.74-1.03)
Hypertension, Pulmonary	1.12 (1.00-1.25)	1.09 (0.88-1.42)	1.03 (0.79-1.03)
Inflammatory Bowel Disease	1.10 (1.02-1.19)	0.97 (0.82-1.10)	1.09 (0.93-1.29)
Knee arthroplasty	1.12 (1.02-1.25)	1.06 (0.86-1.35)	0.99 (0.79-1.26)
Migraine	2.08 (0.97-1.16)	1.02 (0.87-1.18)	1.04 (0.85-1.31)
Osteoarthritis	1.07 (0.94-1.17)	1.05 (0.85-1.29)	1.00 (0.80-1.24)
Osteoporosis	1.12 (1.02-1.24)	1.09 (0.91-1.38)	1.03 (0.82-1.34)
Parkinson’s Disease (all)	1.10 (0.99-1.20)	1.04 (0.83-1.26)	0.94 (0.71-1.16)
Psoriasis	1.10 (0.97-1.24)	1.07 (0.85-1.37)	1.00 (0.75-1.33)
Psoriatic arthropathy	1.11 (0.99-1.25)	1.08 (0.88-1.34)	0.96 (0.73-1.21)
Pulmonary fibrosis	1.09 (0.99-1.19)	1.18 (0.97-1.69)	1.08 (0.87-1.44)
Restless legs syndrome	1.09 (0.98-1.19)	0.98 (0.76-1.15)	1.03 (0.82-1.31)
Rheumatoid arthritis	1.28 (1.08-1.30)	1.08 (0.95-1.23)	1.09 (0.93-1.30)
Thromboembolism	1.10 (1.03-1.17)	1.07 (0.94-1.22)	0.96 (0.85-1.07)

1 - Trial level models adjusted for age, sex and comorbidity count. 2 - Trial level models adjusted for age and sex.

Lees JS, *et al. BMJMED* 2022; 1:e000217. doi: 10.1136/bmjmed-2022-000217

Table S2 Odds ratio for association between attrition and age, sex and comorbidity count by ATC drug class

ATC drug class	Drug type	Comorbidity count[1]	Age[2]	Sex[2]
A10BH	Dipeptidyl peptidase 4 (DPP-4) inhibitors	1.08 (0.98-1.16)	1.08 (0.94-1.29)	0.95 (0.81-1.09)
A10BJ	Glucagon-like peptide-1 receptor analogues	1.09 (1.00-1.16)	1.02 (0.86-1.14)	1.07 (0.92-1.28)
A10BK	Sodium-glucose co-transporter 2 inhibitors	1.09 (1.03-1.16)	1.02 (0.89-1.15)	0.96 (0.84-1.09)
B01AB	Heparin group	1.10 (0.97-1.24)	1.06 (0.90-1.29)	1.06 (0.90-1.26)
B01AE	Direct thrombin inhibitors	1.11 (1.05-1.17)	1.08 (0.96-1.23)	0.92 (0.80-1.04)
C09CA	Angiotensin II antagonists	1.12 (1.04-1.21)	1.06 (0.94-1.22)	0.90 (0.76-1.03)
G04BE	Drugs used in erectile dysfunction	1.13 (1.04-1.26)	1.10 (0.96-1.35)	1.03 (0.80-1.36)
H05AA	Parathyroid hormones and analogues	1.11 (1.01-1.23)	1.07 (0.91-1.31)	1.04 (0.83-1.33)
L01XE	Protein kinase inhibitors	1.09 (0.99-1.18)	1.12 (0.95-1.49)	1.08 (0.88-1.40)
L04AA	Selective immunosuppressants	1.11 (1.01-1.22)	1.03 (0.84-1.19)	1.07 (0.89-1.34)
L04AB	Tumour necrosis factors alpha inhibitors	1.11 (1.04-1.19)	1.01 (0.88-1.12)	1.06 (0.91-1.25)
L04AC	Interleukin inhibitors	1.17 (1.08-1.31)	1.06 (0.95-1.21)	1.07 (0.92-1.27)
M01AC	Oxicams	1.07 (0.93-1.17)	1.05 (0.88-1.25)	1.01 (0.82-1.25)
N03AX	Other antiepileptics	1.08 (0.97-1.16)	1.03 (0.87-1.16)	1.04 (0.85-1.30)
N04BC	Dopamine agonists	1.08 (0.98-1.16)	1.00 (0.81-1.13)	0.98 (0.81-1.17)
N06DA	Anticholinesterase drugs	1.09 (1.01-1.18)	1.06 (0.91-1.26)	0.89 (0.73-1.06)
R03DX	Other systemic drugs for obstructive airway diseases	1.08 (0.95-1.17)	0.99 (0.76-1.13)	1.00 (0.78-1.26)

1 - Trial level models adjusted for age, sex and comorbidity count. 2 - Trial level models adjusted for age and sex.

Lees JS, *et al. BMJMED* 2022; 1:e000217. doi: 10.1136/bmjmed-2022-000217

Table S3 Sensitivity analysis with wider priors on all of the variance components (trial, drug class and index condition)

	**Original priors[1]**	**Wider priors[2]**
**Partial pooling across index condition and drug class**	N=20 index conditions; N=17 drug classes	N=20 index conditions; N=17 drug classes
Intercept (SD)	0.097 (0.026)	0.096 (0.025)
SD trial (SD)	0.091 (0.018)	0.092 (0.018)
SD index condition (SD)	0.044 (0.027)	0.043 (0.027)
SD drug class (SD)	0.036 (0.026)	0.035 (0.026)

1 - Trial level models adjusted for age, sex and comorbidity count using original priors. 2 - Trial level models adjusted for age, sex and comorbidity count using wider priors for variance components. SD: standard deviations (and their SDs) for within-group variation for trials, index conditions and drug classes on log-odds scale. See supplementary methods below for full description of models and selection of priors.

Lees JS, *et al. BMJMED* 2022; 1:e000217. doi: 10.1136/bmjmed-2022-000217

Figure S1

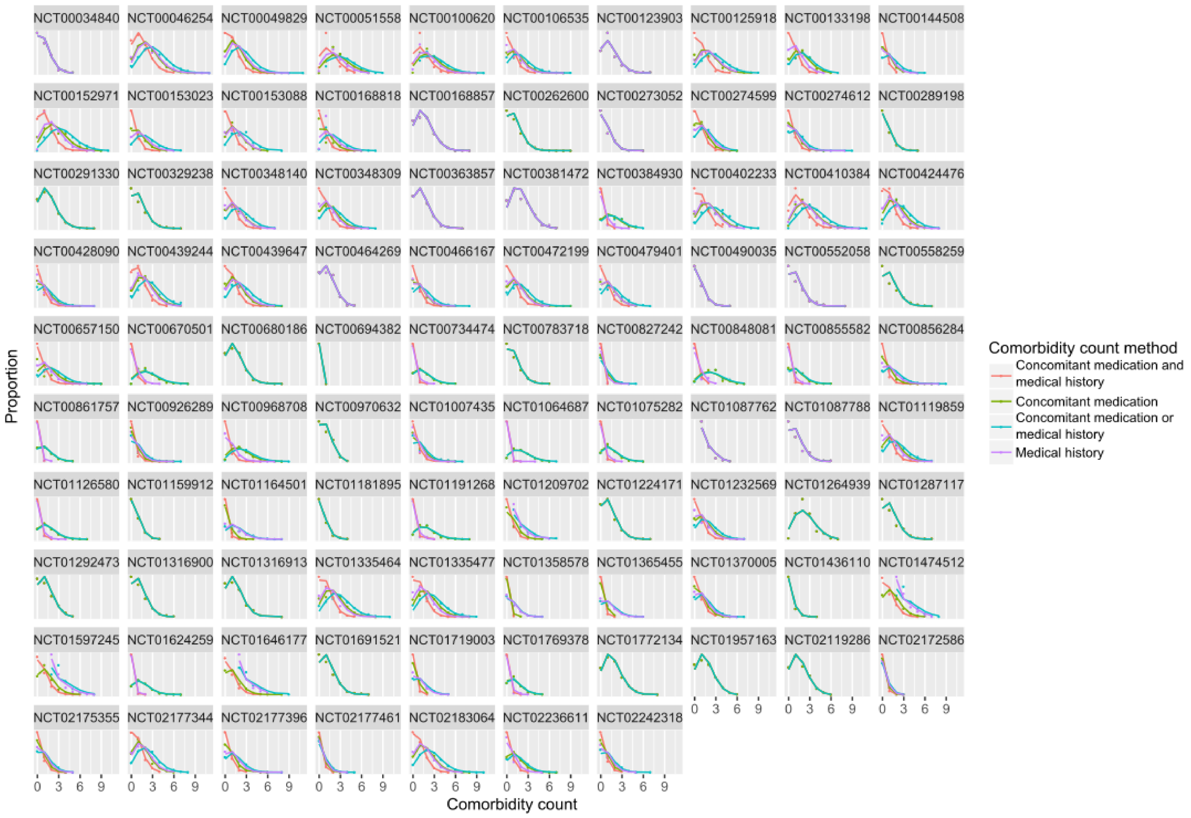

Distribution of comorbidity count for each trial. Points indicate raw proportions. Lines indicate expected proportion under Poisson. The y-axis has been deliberately omitted and the resolution kept low to maintain privacy. The purpose of this plot is to show that the comorbidity counts follow a broadly Poisson distribution as justification for presenting mean counts as summary statistics and to explain the modelling choices used in the analysis of the impact of increasing comorbidity on overall trial attrition.

Detailed description of modelling

Model fitting

For each trial, we fitted generalised linear models to individual-level participant data within each trial repository.

$$inv.logit(p_i) = \beta_0 + \beta_1 \cdot age + \beta_2 \cdot sex + \beta_3 \cdot comorbidity_i$$

Where p_i was the probability of attrition and the β parameters 0, 1, 2 and 3 were the coefficients for the intercept, age, sex and comorbidity count respectively. i indicates the individual within each trial. A model was fitted separately for each of the j trials (subscript not shown above for clarity.) For each trial, we exported the resultant model coefficients (β_0, β_1, β_2, β_3) standard errors (se_{β_0}, se_{β_1}, se_{β_2}, se_{β_3}), and variance-covariance matrices for subsequent meta-analysis. The variance-covariance matrices had been exported in case more complex models needed to be fitted. However, as there was no evidence of departure from linearity, these were not used in any of the subsequent analysis, but are available on our github repository in case they are useful for other researchers.

The following shows the meta analysis for comorbidity for the most complex model shown in Table 2. The analyses for age and sex were similar. The simpler models excluded, respectively, the condition related terms, drug class related terms or both. The model had a normal likelihood and linear predictor as follows:-

$$\beta_{3,j} \sim N\left(\theta_j, se_{beta_{3,j}}\right)$$

$$\theta_j = \alpha_0 + trial_j + cond_k + drug_l$$

α_0 indicates the overall effect. Trial, cond and drug indicate the trial-level, condition-level and drug-level random effects for the j-trials, k-conditions and l-drug classes. Note that trials were nested within drug classes and conditions, but conditions and drug classes were cross-classified (some of the drugs used to treat inflammatory conditions were used across multiple conditions).

The variation in the comorbidity-attrition association at the trial, condition and drug class effects was assumed to be normally distributed (random effects):-

$$trial_j \sim N(\mu_{trial}, \sigma_{trial})$$

$$cond_k \sim N(\mu_{cond}, \sigma_{cond})$$

$$drug_l \sim N(\mu_{drug}, \sigma_{drug})$$

The prior for the overall intercept α was t-distributed with 3 degrees of freedom:-

$$\alpha_0 \sim student\text{-}t(df = 3, mean = 0, sd = 100)$$

For the trial, condition and drug-level random effects, the priors for mean and standard deviation were t-distributed and half-t distributed respectively:-

$$\mu_{trial} \sim student\text{-}t(df = 3, mean = 0, sd = 2.5)$$

$$\mu_{cond} \sim student\text{-}t(df = 3, mean = 0, sd = 2.5)$$

$$\mu_{drug} \sim student\text{-}t(df = 3, mean = 0, sd = 2.5)$$

Lees JS, *et al. BMJMED* 2022; 1:e000217. doi: 10.1136/bmjmed-2022-000217

$$\sigma_{trial} \sim half\text{-}student\text{-}t(df = 3, mean = 0, sd = 2.5)$$

$$\sigma_{cond} \sim half\text{-}student\text{-}t(df = 3, mean = 0, sd = 2.5)$$

$$\sigma_{drug} \sim half\text{-}student\text{-}t(df = 3, mean = 0, sd = 2.5)$$

As a sensitivity analysis of the effect of the selected priors, the models were re-run using wider priors with half-t distribution as follows:

$$\sigma_{trial} \sim half\text{-}student\text{-}t(df = 3, mean = 0, sd = 10)$$

$$\sigma_{cond} \sim half\text{-}student\text{-}t(df = 3, mean = 0, sd = 10)$$

$$\sigma_{drug} \sim half\text{-}student\text{-}t(df = 3, mean = 0, sd = 10)$$

e^{α_0} was the overall estimate for the odds ratio (OR) across all drugs and conditions. The condition-level and drug-level odds ratios were estimated as follows using samples from the posterior:-

$$OR_{cond_k} = e^{\alpha_0 + cond_k}$$

$$OR_{drug_l} = e^{\alpha_0 + drug_l}$$

For all parameters the mean of the un-transformed distribution was obtained and this was subsequently exponentiated to obtain the point estimate for the OR. The credible intervals were obtained as the 2.5th and 97.5th, 10th and 90th and 25th and 75th percentiles.

Application to notional trial-level data

The point estimate for the OR was applied to simulated trial data as follows:-

1. For a set of 5 mean comorbidity counts (0.5, 0.75, 1, 1.25, 2) the probability mass function was used to calculate the proportion of participants with 0, 1, 2 … 100 comorbidities.
2. For each of the above 5 sets of comorbidity counts, the risk of attrition in individuals with zero comorbidities was set to 0.05, 0.15, 0.25, 0.35 and 0.45, giving a total of 25 notional trials (5 mean comorbidity counts X 5 baseline risks of attrition).
3. Each baseline attrition risk was transformed to obtain the baseline odds of attrition.
4. For each comorbidity count (0 to 100), we estimated the odds ratio as $OR^{comorbidity\ count}$. For example, $1.1^0 = 1$, $1.1^1 = 1.1$, $1.1^2 = 1.21$ for comorbidity counts 0, 1 and 2.
5. For each comorbidity count at each level of baseline risk, we estimated the comorbidity-count specific odds of attrition by multiplying the comorbidity-count specific odds ratio (step 4) by the baseline odds of attrition (step 3).
6. We transformed each of the odds from step 5 to obtain the comorbidity-count/baseline risk specific risk of attrition.
7. For each of the 25 notional trials, we calculated a weighted mean of the comorbidity-count specific risk of attrition, where the weights were the probabilities obtained in step 1. This is the trial-level risk of attrition.

The assumptions of this approach are as follows:-

1. Trial comorbidity is Poisson distributed.

2. The odds ratios are applicable to a set of notional trials with these levels for comorbidity count and baseline risk of attrition.
